# *tert*-Butyl 2-(6-{2-[2-(4-fluoro­phen­yl)-5-isopropyl-3-phenyl-4-(phenyl­carbamo­yl)pyrrol-1-yl]eth­yl}-2,2-dimethyl-1,3-dioxan-4-yl)acetate

**DOI:** 10.1107/S160053681302624X

**Published:** 2013-10-09

**Authors:** Ya-Ming Wu

**Affiliations:** a Department of Applied Chemistry, Nanjing College of Chemical Technology, No. 625 Geguan Road, Dachang, Nanjing 210048, People’s Republic of China

## Abstract

The title compound, C_40_H_47_FN_2_O_5_, crystallizes with two independent but similar mol­ecules in the asymmetric unit. In the crystal, mol­ecules are linked into chains along [100] by inter­molecular N—H⋯O hydrogen bonds.

## Related literature

For applications of the title compound, see: Zhang *et al.* (2012 ▶). For the synthesis, see: Zhang *et al.* (2012 ▶). For standard bond lengths, see: Allen *et al.* (1987 ▶).
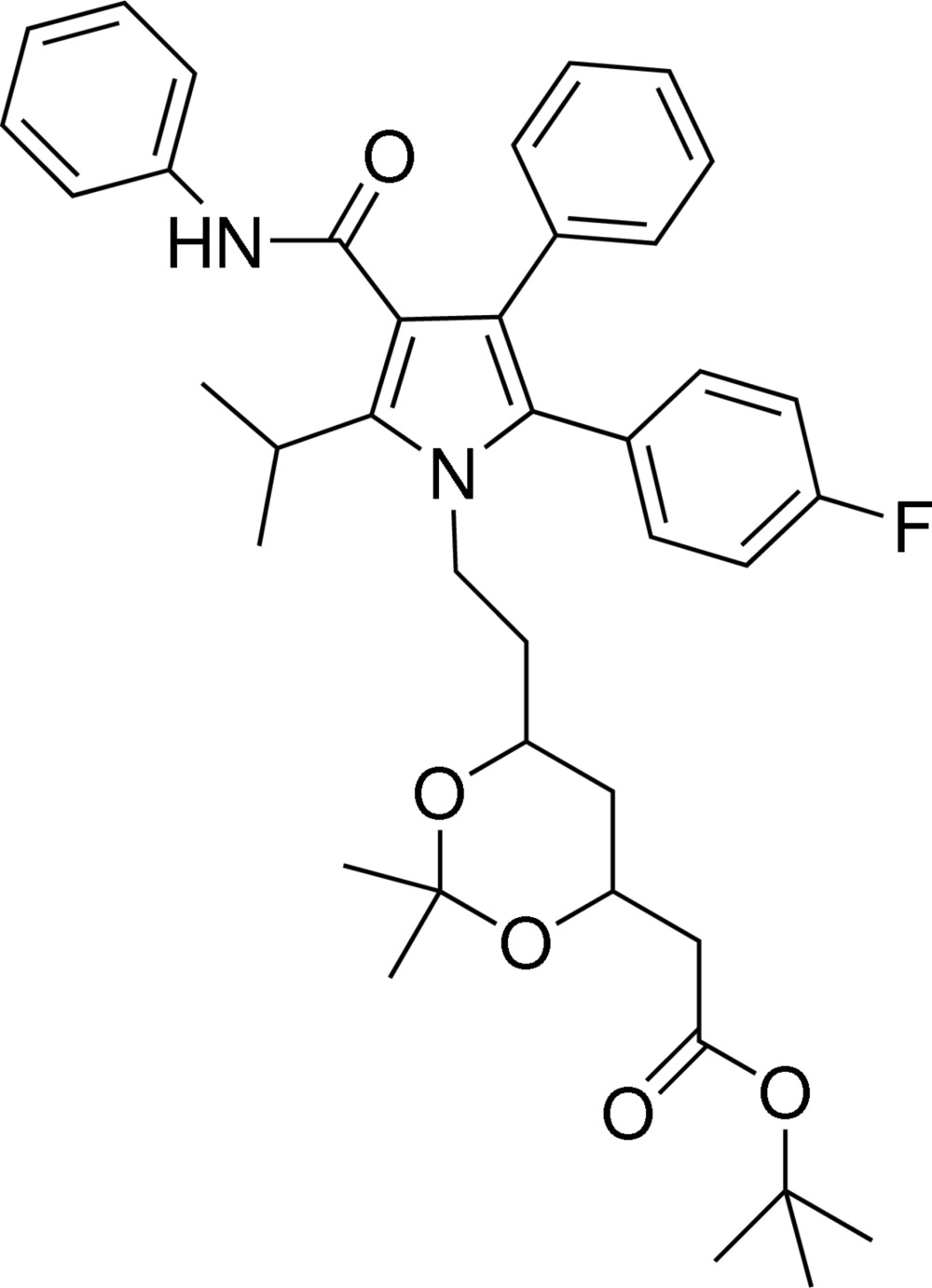


## Experimental

### 

#### Crystal data

C_40_H_47_FN_2_O_5_
*M* *_r_* = 654.79
Monoclinic, 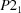

*a* = 13.439 (3) Å
*b* = 15.636 (3) Å
*c* = 18.644 (4) Å
β = 108.60 (3)°
*V* = 3713.1 (13) Å^3^
*Z* = 4
Mo *K*α radiation
μ = 0.08 mm^−1^
*T* = 293 K
0.30 × 0.20 × 0.10 mm

#### Data collection

Enraf–Nonius CAD-4 diffractometer
Absorption correction: ψ scan (North *et al.*, 1968 ▶) *T*_min_ = 0.976, *T*_max_ = 0.992
7418 measured reflections
7100 independent reflections
3663 reflections with *I* > 2σ(*I*)
*R*_int_ = 0.099
3 standard reflections every 200 reflections intensity decay: 1%

#### Refinement

*R*[*F* ^2^ > 2σ(*F* ^2^)] = 0.059
*wR*(*F* ^2^) = 0.104
*S* = 1.00
7100 reflections
865 parameters
1 restraint
H-atom parameters constrained
Δρ_max_ = 0.19 e Å^−3^
Δρ_min_ = −0.22 e Å^−3^

### 

Data collection: *CAD-4 EXPRESS* (Enraf–Nonius, 1994 ▶); cell refinement: *CAD-4 EXPRESS*; data reduction: *XCAD4* (Harms & Wocadlo, 1995 ▶); program(s) used to solve structure: *SHELXS97* (Sheldrick, 2008 ▶); program(s) used to refine structure: *SHELXL97* (Sheldrick, 2008 ▶); molecular graphics: *SHELXTL* (Sheldrick, 2008 ▶); software used to prepare material for publication: *SHELXTL*.

## Supplementary Material

Crystal structure: contains datablock(s) I, New_Global_Publ_Block. DOI: 10.1107/S160053681302624X/vm2197sup1.cif

Structure factors: contains datablock(s) I. DOI: 10.1107/S160053681302624X/vm2197Isup2.hkl

Supplementary material file. DOI: 10.1107/S160053681302624X/vm2197Isup3.cml

Additional supplementary materials:  crystallographic information; 3D view; checkCIF report

## Figures and Tables

**Table 1:** Hydrogen-bond geometry (Å, °)

*D*—H⋯*A*	*D*—H	H⋯*A*	*D*⋯*A*	*D*—H⋯*A*
N2—H2*A*⋯O2^i^	0.86	2.05	2.899 (7)	168
N4—H4*A*⋯O7^ii^	0.86	2.07	2.914 (7)	168

Symmetry codes: (i) 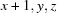; (ii) 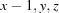.

**Fig. 1.**
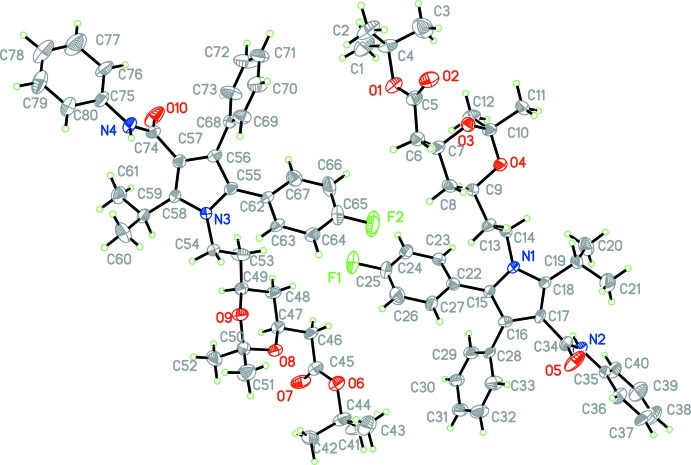
The molecular structure of (I), with the atom-numbering scheme. Displacement ellipsoids are drawn at the 50% probability level.

**Fig. 2.**
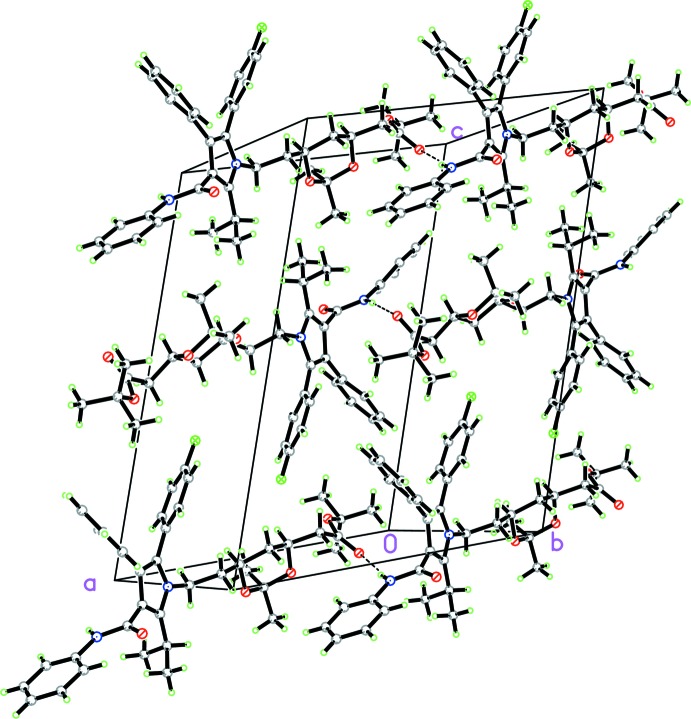
Packing diagram of (I) showing N—H···O hydrogen bonds as broken lines.

## supplementary crystallographic information

### 1. Comment

The title compound, (I), is an intermediate for the preparation of atorvastatin (Zhang *et al.*, 2012). We herein report its molecular and crystal structure (Fig. 1). The asymmetric unit of the title compound, C_40_H_47_FN_2_O_5_, contains two independent molecules with similar structure (r.m.s. deviation 0.357 Å). The bond lengths and angles are within normal ranges (Allen *et al.*, 1987). The dihedral angles between the aromatic rings are: 52.1 (3)° (A/B), 51.2 (3)° (A/C), 86.5 (3)° (A,D), 48.3 (3)° (E/F), 57.1 (3)° (E/G) and 54.4 (4)° (E,H) [with the rings defined as: A =N1/C15—C18, B=C22—C27, C=C28—C33, D=C35—C40, E=N3/C55—C58, F=C62—C67, G=C68—C73 and H=C75—C80].

In the crystal packing, the molecules are linked into chains by intermolecular N—H···O hydrogen bonds (Fig. 2 and Table 1).

### 2. Experimental

The title compound was synthesized according to a procedure published by Zhang *et al.* (2012). Crystals suitable for X-ray analysis were obtained by dissolving the compound (0.5 g) in ethanol (80 ml) and evaporating the solvent slowly at room temperature for about 5 d.

### 3. Refinement

H atoms were positioned geometrically, with C—H = 0.93, 0.96, 0.97 and 0.98 Å for aromatic, methyl, methylene and methine H, respectively, and N—H = 0.86 Å and constrained to ride on their parent atoms, with *U*_iso_(H) = *xU*_eq_(C/N), where *x* = 1.5 for methyl H and *x* = 1.2 for other H atoms.

### Crystal data

**Table:**

C_40_H_47_FN_2_O_5_	*F*(000) = 1400
*M**_r_* = 654.79	*D*_x_ = 1.171 Mg m^−^^3^
Monoclinic, *P*2_1_	Mo *K*α radiation, λ = 0.71073 Å
Hall symbol: P 2yb	Cell parameters from 25 reflections
*a* = 13.439 (3) Å	θ = 9–13°
*b* = 15.636 (3) Å	µ = 0.08 mm^−^^1^
*c* = 18.644 (4) Å	*T* = 293 K
β = 108.60 (3)°	Block, white
*V* = 3713.1 (13) Å^3^	0.30 × 0.20 × 0.10 mm
*Z* = 4	

### Data collection

**Table:**

Enraf–Nonius CAD-4 diffractometer	3663 reflections with *I* > 2σ(*I*)
Radiation source: fine-focus sealed tube	*R*_int_ = 0.099
Graphite monochromator	θ_max_ = 25.4°, θ_min_ = 1.2°
ω/2θ scans	*h* = 0→16
Absorption correction: ψ scan (North *et al.*, 1968)	*k* = 0→18
*T*_min_ = 0.976, *T*_max_ = 0.992	*l* = −22→21
7418 measured reflections	3 standard reflections every 200 reflections
7100 independent reflections	intensity decay: 1%

### Refinement

**Table:**

Refinement on *F*^2^	Primary atom site location: structure-invariant direct methods
Least-squares matrix: full	Secondary atom site location: difference Fourier map
*R*[*F*^2^ > 2σ(*F*^2^)] = 0.059	Hydrogen site location: inferred from neighbouring sites
*wR*(*F*^2^) = 0.104	H-atom parameters constrained
*S* = 1.00	*w* = 1/[σ^2^(*F*_o_^2^) + (0.026*P*)^2^] where *P* = (*F*_o_^2^ + 2*F*_c_^2^)/3
7100 reflections	(Δ/σ)_max_ < 0.001
865 parameters	Δρ_max_ = 0.19 e Å^−^^3^
1 restraint	Δρ_min_ = −0.22 e Å^−^^3^

### Special details

**Table:**

Geometry. All e.s.d.'s (except the e.s.d. in the dihedral angle between two l.s. planes) are estimated using the full covariance matrix. The cell e.s.d.'s are taken into account individually in the estimation of e.s.d.'s in distances, angles and torsion angles; correlations between e.s.d.'s in cell parameters are only used when they are defined by crystal symmetry. An approximate (isotropic) treatment of cell e.s.d.'s is used for estimating e.s.d.'s involving l.s. planes.
Refinement. Refinement of *F*^2^ against ALL reflections. The weighted *R*-factor *wR* and goodness of fit *S* are based on *F*^2^, conventional *R*-factors *R* are based on *F*, with *F* set to zero for negative *F*^2^. The threshold expression of *F*^2^ > σ(*F*^2^) is used only for calculating *R*-factors(gt) *etc*. and is not relevant to the choice of reflections for refinement. *R*-factors based on *F*^2^ are statistically about twice as large as those based on *F*, and *R*- factors based on ALL data will be even larger.

### Fractional atomic coordinates and isotropic or equivalent isotropic displacement parameters (Å^2^)

**Table:**

	*x*	*y*	*z*	*U*_iso_*/*U*_eq_	
O1	0.3130 (3)	0.1418 (3)	0.0666 (2)	0.0597 (12)	
O2	0.2185 (3)	0.2258 (3)	−0.0277 (3)	0.0654 (13)	
O3	0.4393 (3)	0.1809 (3)	−0.04862 (19)	0.0487 (10)	
O4	0.5975 (3)	0.1890 (3)	−0.07450 (19)	0.0478 (10)	
O5	1.0683 (4)	0.5083 (3)	−0.0990 (3)	0.0874 (17)	
F1	0.8952 (4)	0.2611 (4)	0.3190 (2)	0.135 (2)	
N1	0.9191 (3)	0.2897 (3)	−0.0191 (2)	0.0423 (12)	
N2	1.1740 (3)	0.3936 (3)	−0.0966 (2)	0.0478 (13)	
H2A	1.1843	0.3410	−0.0829	0.057*	
C1	0.1358 (6)	0.1115 (5)	0.0672 (5)	0.094 (3)	
H1A	0.1553	0.1413	0.1147	0.141*	
H1B	0.0874	0.0665	0.0678	0.141*	
H1C	0.1032	0.1506	0.0269	0.141*	
C2	0.2826 (6)	0.0125 (5)	0.1190 (4)	0.100 (3)	
H2B	0.2958	0.0408	0.1668	0.150*	
H2C	0.3478	−0.0069	0.1139	0.150*	
H2D	0.2373	−0.0356	0.1164	0.150*	
C3	0.2115 (7)	0.0333 (5)	−0.0202 (4)	0.103 (3)	
H3A	0.1792	0.0738	−0.0594	0.154*	
H3B	0.1661	−0.0152	−0.0247	0.154*	
H3C	0.2772	0.0148	−0.0251	0.154*	
C4	0.2304 (5)	0.0744 (5)	0.0554 (4)	0.061 (2)	
C5	0.2986 (5)	0.2099 (5)	0.0224 (4)	0.0535 (17)	
C6	0.3943 (4)	0.2655 (4)	0.0431 (3)	0.0546 (17)	
H6A	0.4148	0.2799	0.0964	0.066*	
H6B	0.3792	0.3181	0.0141	0.066*	
C7	0.4842 (4)	0.2174 (4)	0.0262 (3)	0.0528 (15)	
H7A	0.5098	0.1715	0.0633	0.063*	
C8	0.5747 (4)	0.2756 (4)	0.0265 (3)	0.0616 (19)	
H8A	0.6095	0.2963	0.0774	0.074*	
H8B	0.5485	0.3245	−0.0060	0.074*	
C9	0.6524 (4)	0.2260 (4)	−0.0017 (3)	0.0492 (14)	
H9A	0.6849	0.1806	0.0343	0.059*	
C10	0.5113 (4)	0.1363 (4)	−0.0759 (3)	0.0480 (16)	
C11	0.4506 (5)	0.1194 (4)	−0.1593 (3)	0.068 (2)	
H11A	0.4279	0.1728	−0.1847	0.102*	
H11B	0.3906	0.0844	−0.1630	0.102*	
H11C	0.4954	0.0905	−0.1826	0.102*	
C12	0.5434 (5)	0.0525 (4)	−0.0337 (4)	0.0650 (19)	
H12A	0.5802	0.0641	0.0186	0.098*	
H12B	0.5884	0.0213	−0.0553	0.098*	
H12C	0.4819	0.0192	−0.0377	0.098*	
C13	0.7365 (4)	0.2839 (4)	−0.0124 (3)	0.0539 (17)	
H13A	0.7048	0.3227	−0.0540	0.065*	
H13B	0.7654	0.3181	0.0329	0.065*	
C14	0.8245 (4)	0.2362 (4)	−0.0284 (3)	0.0472 (16)	
H14A	0.8438	0.1875	0.0054	0.057*	
H14B	0.7997	0.2145	−0.0798	0.057*	
C15	0.9849 (4)	0.3189 (4)	0.0499 (3)	0.0415 (15)	
C16	1.0570 (4)	0.3730 (4)	0.0355 (3)	0.0425 (15)	
C17	1.0329 (4)	0.3779 (4)	−0.0437 (3)	0.0393 (15)	
C18	0.9474 (4)	0.3272 (4)	−0.0769 (3)	0.0426 (15)	
C19	0.8945 (5)	0.3015 (5)	−0.1576 (3)	0.0567 (19)	
H19A	0.8194	0.2949	−0.1650	0.068*	
C20	0.9351 (6)	0.2165 (5)	−0.1756 (4)	0.088 (3)	
H20A	0.9269	0.1738	−0.1409	0.132*	
H20B	1.0081	0.2218	−0.1710	0.132*	
H20C	0.8961	0.1999	−0.2264	0.132*	
C21	0.9067 (6)	0.3704 (6)	−0.2135 (3)	0.094 (3)	
H21A	0.8732	0.3513	−0.2645	0.141*	
H21B	0.9799	0.3802	−0.2058	0.141*	
H21C	0.8745	0.4227	−0.2051	0.141*	
C22	0.9649 (4)	0.3015 (4)	0.1220 (3)	0.0466 (15)	
C23	0.9530 (4)	0.2195 (4)	0.1472 (3)	0.0510 (16)	
H23A	0.9622	0.1727	0.1192	0.061*	
C24	0.9282 (5)	0.2059 (5)	0.2119 (3)	0.0624 (19)	
H24A	0.9172	0.1508	0.2267	0.075*	
C25	0.9201 (6)	0.2743 (6)	0.2539 (4)	0.076 (2)	
C26	0.9361 (6)	0.3550 (5)	0.2344 (4)	0.085 (3)	
H26A	0.9317	0.4006	0.2654	0.102*	
C27	0.9590 (5)	0.3699 (5)	0.1683 (3)	0.0634 (19)	
H27A	0.9703	0.4254	0.1547	0.076*	
C28	1.1479 (4)	0.4110 (4)	0.0935 (3)	0.0413 (15)	
C29	1.2151 (5)	0.3607 (5)	0.1487 (3)	0.0600 (19)	
H29A	1.2021	0.3023	0.1487	0.072*	
C30	1.3012 (5)	0.3941 (5)	0.2041 (3)	0.0617 (19)	
H30A	1.3442	0.3585	0.2410	0.074*	
C31	1.3229 (5)	0.4797 (5)	0.2044 (4)	0.064 (2)	
H31A	1.3797	0.5031	0.2419	0.077*	
C32	1.2592 (6)	0.5301 (5)	0.1483 (4)	0.076 (2)	
H32A	1.2744	0.5879	0.1465	0.091*	
C33	1.1727 (5)	0.4959 (4)	0.0944 (4)	0.066 (2)	
H33A	1.1299	0.5317	0.0576	0.079*	
C34	1.0917 (5)	0.4326 (4)	−0.0816 (3)	0.0473 (16)	
C35	1.2448 (5)	0.4279 (4)	−0.1317 (3)	0.0461 (16)	
C36	1.2423 (5)	0.5113 (5)	−0.1550 (3)	0.065 (2)	
H36A	1.1927	0.5488	−0.1475	0.078*	
C37	1.3131 (6)	0.5401 (5)	−0.1895 (4)	0.083 (2)	
H37A	1.3108	0.5969	−0.2047	0.099*	
C38	1.3866 (6)	0.4863 (6)	−0.2018 (5)	0.092 (3)	
H38A	1.4332	0.5055	−0.2260	0.111*	
C39	1.3895 (6)	0.4029 (6)	−0.1773 (4)	0.098 (3)	
H39A	1.4394	0.3657	−0.1845	0.118*	
C40	1.3187 (5)	0.3728 (4)	−0.1416 (3)	0.0638 (17)	
H40A	1.3220	0.3165	−0.1249	0.077*	
F2	0.6114 (4)	0.3625 (4)	0.1940 (2)	0.1283 (19)	
O6	1.2025 (3)	0.4521 (3)	0.4415 (2)	0.0669 (13)	
O7	1.2878 (4)	0.3828 (3)	0.5494 (3)	0.0745 (14)	
O8	1.0404 (3)	0.4435 (3)	0.5251 (2)	0.0538 (11)	
O9	0.8805 (3)	0.4336 (3)	0.5485 (2)	0.0569 (12)	
O10	0.4265 (4)	0.0903 (3)	0.5996 (3)	0.0900 (18)	
N3	0.5953 (3)	0.3064 (3)	0.5323 (2)	0.0428 (12)	
N4	0.3355 (4)	0.2088 (3)	0.6058 (2)	0.0492 (13)	
H4A	0.3292	0.2623	0.5944	0.059*	
C41	1.3828 (6)	0.4827 (5)	0.4474 (6)	0.117 (3)	
H41A	1.4120	0.4453	0.4897	0.176*	
H41B	1.4316	0.5279	0.4485	0.176*	
H41C	1.3692	0.4511	0.4011	0.176*	
C42	1.2913 (6)	0.5694 (5)	0.5232 (4)	0.082 (2)	
H42A	1.3223	0.5332	0.5661	0.123*	
H42B	1.2232	0.5877	0.5233	0.123*	
H42C	1.3352	0.6185	0.5257	0.123*	
C43	1.2291 (7)	0.5774 (6)	0.3827 (4)	0.119 (3)	
H43A	1.1636	0.5990	0.3855	0.178*	
H43B	1.2167	0.5443	0.3373	0.178*	
H43C	1.2748	0.6244	0.3820	0.178*	
C44	1.2807 (6)	0.5207 (5)	0.4518 (5)	0.075 (2)	
C45	1.2134 (5)	0.3901 (4)	0.4930 (4)	0.0551 (18)	
C46	1.1176 (5)	0.3347 (4)	0.4751 (3)	0.0635 (19)	
H46A	1.1383	0.2750	0.4793	0.076*	
H46B	1.0734	0.3451	0.4235	0.076*	
C47	1.0569 (4)	0.3533 (4)	0.5286 (3)	0.0492 (14)	
H47A	1.0990	0.3370	0.5801	0.059*	
C48	0.9528 (4)	0.3085 (4)	0.5073 (3)	0.0550 (17)	
H48A	0.9642	0.2476	0.5162	0.066*	
H48B	0.9164	0.3168	0.4538	0.066*	
C49	0.8848 (4)	0.3417 (4)	0.5527 (3)	0.0536 (15)	
H49A	0.9136	0.3233	0.6056	0.064*	
C50	0.9811 (5)	0.4750 (5)	0.5716 (4)	0.0564 (18)	
C51	0.9610 (6)	0.5689 (5)	0.5535 (4)	0.087 (2)	
H51A	0.9225	0.5756	0.5008	0.130*	
H51B	1.0268	0.5985	0.5650	0.130*	
H51C	0.9211	0.5922	0.5834	0.130*	
C52	1.0377 (5)	0.4627 (5)	0.6544 (3)	0.088 (3)	
H52A	1.0511	0.4029	0.6648	0.133*	
H52B	0.9951	0.4841	0.6832	0.133*	
H52C	1.1031	0.4933	0.6683	0.133*	
C53	0.7731 (4)	0.3105 (4)	0.5189 (4)	0.067 (2)	
H53A	0.7525	0.3171	0.4644	0.080*	
H53B	0.7714	0.2499	0.5295	0.080*	
C54	0.6933 (4)	0.3548 (4)	0.5465 (3)	0.0475 (16)	
H54A	0.7234	0.3648	0.6005	0.057*	
H54B	0.6774	0.4101	0.5219	0.057*	
C55	0.5303 (4)	0.2801 (4)	0.4605 (3)	0.0438 (15)	
C56	0.4548 (4)	0.2267 (4)	0.4725 (3)	0.0434 (15)	
C57	0.4755 (4)	0.2202 (4)	0.5526 (3)	0.0456 (16)	
C58	0.5604 (4)	0.2695 (4)	0.5878 (3)	0.0426 (15)	
C59	0.6099 (5)	0.2903 (4)	0.6695 (3)	0.0544 (17)	
H59A	0.6856	0.2952	0.6785	0.065*	
C60	0.5715 (6)	0.3788 (5)	0.6877 (4)	0.098 (3)	
H60A	0.5829	0.4210	0.6538	0.146*	
H60B	0.6097	0.3943	0.7389	0.146*	
H60C	0.4979	0.3757	0.6817	0.146*	
C61	0.5949 (5)	0.2228 (5)	0.7231 (3)	0.080 (2)	
H61A	0.6283	0.2410	0.7743	0.120*	
H61B	0.6255	0.1700	0.7143	0.120*	
H61C	0.5212	0.2147	0.7146	0.120*	
C62	0.5473 (4)	0.3040 (4)	0.3898 (3)	0.0438 (15)	
C63	0.5648 (4)	0.3885 (4)	0.3714 (3)	0.0481 (15)	
H63A	0.5609	0.4322	0.4042	0.058*	
C64	0.5872 (5)	0.4090 (5)	0.3073 (3)	0.0617 (19)	
H64A	0.6007	0.4653	0.2970	0.074*	
C65	0.5893 (6)	0.3445 (6)	0.2586 (4)	0.077 (2)	
C66	0.5680 (6)	0.2596 (6)	0.2715 (4)	0.081 (2)	
H66A	0.5680	0.2170	0.2367	0.097*	
C67	0.5470 (5)	0.2406 (5)	0.3373 (3)	0.064 (2)	
H67A	0.5324	0.1844	0.3469	0.076*	
C68	0.3636 (5)	0.1904 (4)	0.4129 (3)	0.0460 (16)	
C69	0.2946 (5)	0.2440 (4)	0.3621 (3)	0.0582 (19)	
H69A	0.3052	0.3028	0.3663	0.070*	
C70	0.2103 (5)	0.2119 (5)	0.3053 (4)	0.073 (2)	
H70A	0.1664	0.2491	0.2703	0.088*	
C71	0.1900 (5)	0.1250 (5)	0.2995 (4)	0.069 (2)	
H71A	0.1328	0.1033	0.2613	0.083*	
C72	0.2570 (5)	0.0709 (5)	0.3520 (4)	0.088 (3)	
H72A	0.2442	0.0124	0.3490	0.106*	
C73	0.3423 (6)	0.1024 (4)	0.4086 (4)	0.075 (2)	
H73A	0.3857	0.0654	0.4440	0.090*	
C74	0.4107 (5)	0.1659 (4)	0.5892 (3)	0.0488 (16)	
C75	0.2655 (5)	0.1746 (5)	0.6406 (3)	0.0581 (19)	
C76	0.2330 (5)	0.0916 (5)	0.6325 (4)	0.079 (2)	
H76A	0.2575	0.0541	0.6032	0.095*	
C77	0.1621 (8)	0.0630 (7)	0.6688 (6)	0.130 (4)	
H77A	0.1370	0.0071	0.6622	0.156*	
C78	0.1306 (8)	0.1185 (8)	0.7140 (6)	0.129 (4)	
H78A	0.0854	0.0992	0.7394	0.155*	
C79	0.1634 (6)	0.2008 (7)	0.7228 (4)	0.099 (3)	
H79A	0.1389	0.2378	0.7526	0.119*	
C80	0.2318 (5)	0.2296 (4)	0.6884 (3)	0.0667 (18)	
H80A	0.2566	0.2854	0.6962	0.080*	

### Atomic displacement parameters (Å^2^)

**Table:**

	*U*^11^	*U*^22^	*U*^33^	*U*^12^	*U*^13^	*U*^23^
O1	0.054 (3)	0.057 (3)	0.076 (3)	−0.001 (2)	0.032 (2)	0.011 (3)
O2	0.052 (3)	0.057 (3)	0.089 (3)	0.001 (2)	0.024 (2)	0.008 (3)
O3	0.045 (2)	0.056 (3)	0.047 (2)	0.000 (2)	0.0183 (19)	−0.005 (2)
O4	0.043 (2)	0.054 (3)	0.052 (2)	−0.008 (2)	0.0231 (18)	−0.007 (2)
O5	0.098 (4)	0.051 (3)	0.143 (5)	0.026 (3)	0.080 (4)	0.045 (3)
F1	0.181 (5)	0.182 (5)	0.072 (3)	−0.043 (4)	0.082 (3)	−0.012 (3)
N1	0.040 (3)	0.049 (3)	0.043 (3)	−0.009 (2)	0.020 (2)	0.002 (3)
N2	0.046 (3)	0.042 (3)	0.063 (3)	0.002 (3)	0.027 (3)	0.008 (3)
C1	0.082 (5)	0.072 (6)	0.158 (8)	−0.004 (5)	0.079 (6)	0.011 (5)
C2	0.111 (7)	0.077 (6)	0.130 (7)	0.003 (5)	0.065 (6)	0.029 (6)
C3	0.129 (8)	0.075 (6)	0.100 (6)	−0.010 (6)	0.028 (6)	−0.010 (6)
C4	0.049 (4)	0.044 (4)	0.094 (5)	−0.009 (4)	0.026 (4)	0.004 (4)
C5	0.046 (4)	0.052 (5)	0.068 (4)	0.002 (4)	0.026 (3)	0.000 (4)
C6	0.052 (4)	0.061 (5)	0.056 (4)	−0.010 (4)	0.024 (3)	−0.010 (3)
C7	0.043 (3)	0.060 (4)	0.062 (4)	−0.007 (3)	0.026 (3)	−0.001 (3)
C8	0.043 (3)	0.085 (5)	0.062 (4)	−0.020 (4)	0.024 (3)	−0.025 (4)
C9	0.043 (3)	0.064 (4)	0.040 (3)	−0.015 (3)	0.013 (3)	−0.005 (3)
C10	0.038 (3)	0.053 (4)	0.057 (4)	−0.005 (3)	0.021 (3)	−0.007 (3)
C11	0.069 (4)	0.085 (5)	0.049 (4)	−0.023 (4)	0.018 (3)	−0.024 (4)
C12	0.066 (4)	0.041 (4)	0.096 (5)	−0.001 (3)	0.037 (4)	−0.007 (4)
C13	0.044 (4)	0.066 (5)	0.062 (4)	−0.015 (3)	0.031 (3)	−0.006 (4)
C14	0.046 (3)	0.055 (4)	0.043 (3)	−0.011 (3)	0.017 (3)	−0.013 (3)
C15	0.043 (3)	0.044 (4)	0.039 (3)	−0.003 (3)	0.016 (3)	0.004 (3)
C16	0.032 (3)	0.038 (4)	0.057 (4)	−0.003 (3)	0.014 (3)	−0.001 (3)
C17	0.039 (3)	0.044 (4)	0.043 (3)	0.008 (3)	0.026 (3)	0.010 (3)
C18	0.046 (4)	0.042 (4)	0.044 (3)	−0.007 (3)	0.020 (3)	−0.002 (3)
C19	0.045 (4)	0.087 (5)	0.041 (3)	−0.015 (4)	0.018 (3)	−0.011 (4)
C20	0.106 (6)	0.099 (7)	0.069 (5)	−0.003 (6)	0.040 (4)	−0.029 (5)
C21	0.092 (6)	0.125 (7)	0.064 (4)	−0.013 (6)	0.021 (4)	0.013 (5)
C22	0.041 (3)	0.049 (4)	0.051 (4)	−0.002 (3)	0.017 (3)	−0.006 (3)
C23	0.051 (4)	0.050 (4)	0.052 (4)	−0.006 (3)	0.016 (3)	−0.005 (3)
C24	0.061 (4)	0.075 (5)	0.056 (4)	−0.010 (4)	0.025 (3)	0.007 (4)
C25	0.092 (5)	0.097 (7)	0.049 (4)	−0.015 (5)	0.037 (4)	0.003 (4)
C26	0.120 (7)	0.071 (6)	0.080 (5)	−0.006 (5)	0.053 (5)	−0.022 (5)
C27	0.091 (5)	0.055 (5)	0.059 (4)	−0.013 (4)	0.045 (4)	−0.006 (4)
C28	0.040 (3)	0.042 (4)	0.049 (3)	−0.010 (3)	0.025 (3)	0.002 (3)
C29	0.048 (4)	0.063 (5)	0.063 (4)	−0.013 (4)	0.010 (3)	0.003 (4)
C30	0.058 (4)	0.058 (5)	0.060 (4)	−0.015 (4)	0.007 (3)	0.008 (4)
C31	0.058 (4)	0.061 (5)	0.064 (5)	−0.013 (4)	0.007 (4)	0.005 (4)
C32	0.079 (5)	0.049 (5)	0.089 (5)	−0.015 (4)	0.011 (5)	0.015 (4)
C33	0.052 (4)	0.057 (5)	0.074 (5)	−0.001 (4)	0.001 (4)	0.015 (4)
C34	0.046 (4)	0.049 (5)	0.052 (4)	0.008 (3)	0.023 (3)	0.010 (3)
C35	0.047 (4)	0.055 (5)	0.038 (3)	−0.009 (3)	0.016 (3)	0.000 (3)
C36	0.063 (4)	0.063 (5)	0.071 (4)	0.003 (4)	0.026 (4)	0.023 (4)
C37	0.075 (5)	0.083 (6)	0.105 (6)	−0.025 (5)	0.050 (5)	0.021 (5)
C38	0.088 (6)	0.106 (8)	0.109 (7)	−0.008 (6)	0.070 (5)	0.021 (6)
C39	0.086 (6)	0.108 (7)	0.132 (7)	0.007 (5)	0.078 (5)	0.009 (6)
C40	0.080 (4)	0.053 (4)	0.077 (4)	0.011 (4)	0.051 (4)	0.003 (4)
F2	0.190 (5)	0.147 (5)	0.079 (3)	−0.012 (4)	0.087 (3)	0.003 (3)
O6	0.061 (3)	0.066 (3)	0.081 (3)	−0.008 (3)	0.033 (2)	0.004 (3)
O7	0.059 (3)	0.048 (3)	0.110 (4)	−0.002 (3)	0.018 (3)	0.022 (3)
O8	0.050 (2)	0.052 (3)	0.063 (3)	−0.014 (2)	0.023 (2)	−0.004 (2)
O9	0.046 (2)	0.051 (3)	0.075 (3)	−0.010 (2)	0.022 (2)	−0.008 (2)
O10	0.105 (4)	0.044 (3)	0.157 (5)	0.011 (3)	0.093 (4)	0.024 (3)
N3	0.043 (3)	0.051 (3)	0.037 (3)	−0.011 (3)	0.016 (2)	−0.007 (2)
N4	0.051 (3)	0.046 (3)	0.061 (3)	0.006 (3)	0.034 (3)	0.008 (3)
C41	0.079 (6)	0.078 (6)	0.231 (10)	−0.012 (5)	0.101 (7)	−0.005 (7)
C42	0.120 (7)	0.043 (5)	0.087 (5)	−0.016 (5)	0.040 (5)	−0.009 (4)
C43	0.159 (9)	0.104 (7)	0.086 (6)	−0.024 (7)	0.029 (6)	0.043 (6)
C44	0.077 (5)	0.047 (5)	0.113 (6)	−0.013 (4)	0.049 (5)	0.005 (5)
C45	0.052 (4)	0.042 (4)	0.081 (5)	0.006 (4)	0.035 (4)	0.007 (4)
C46	0.058 (4)	0.067 (5)	0.080 (4)	−0.021 (4)	0.043 (4)	−0.009 (4)
C47	0.046 (3)	0.047 (4)	0.058 (4)	−0.001 (3)	0.021 (3)	0.004 (3)
C48	0.041 (3)	0.052 (4)	0.076 (4)	−0.006 (3)	0.025 (3)	−0.006 (3)
C49	0.053 (4)	0.051 (4)	0.062 (4)	−0.010 (3)	0.026 (3)	−0.008 (3)
C50	0.054 (4)	0.062 (5)	0.060 (4)	−0.011 (4)	0.028 (3)	−0.014 (4)
C51	0.101 (6)	0.057 (5)	0.122 (6)	−0.007 (4)	0.065 (5)	−0.008 (5)
C52	0.063 (4)	0.128 (7)	0.079 (5)	−0.033 (5)	0.030 (4)	−0.044 (5)
C53	0.046 (4)	0.050 (5)	0.106 (5)	−0.007 (4)	0.028 (4)	−0.022 (4)
C54	0.050 (4)	0.048 (4)	0.049 (3)	−0.015 (3)	0.023 (3)	−0.007 (3)
C55	0.039 (3)	0.042 (4)	0.058 (4)	−0.003 (3)	0.027 (3)	−0.005 (3)
C56	0.039 (3)	0.037 (4)	0.056 (4)	−0.001 (3)	0.019 (3)	0.006 (3)
C57	0.049 (4)	0.042 (4)	0.049 (4)	−0.001 (3)	0.020 (3)	−0.001 (3)
C58	0.039 (3)	0.050 (4)	0.044 (3)	−0.003 (3)	0.022 (3)	−0.001 (3)
C59	0.051 (4)	0.070 (5)	0.047 (4)	−0.011 (4)	0.023 (3)	0.004 (4)
C60	0.120 (7)	0.102 (7)	0.077 (5)	−0.011 (6)	0.041 (5)	−0.040 (5)
C61	0.081 (5)	0.111 (7)	0.047 (4)	−0.017 (5)	0.018 (3)	0.016 (4)
C62	0.042 (3)	0.048 (4)	0.046 (3)	−0.013 (3)	0.020 (3)	−0.002 (3)
C63	0.034 (3)	0.057 (4)	0.059 (4)	−0.007 (3)	0.024 (3)	0.005 (3)
C64	0.067 (4)	0.066 (5)	0.048 (4)	−0.020 (4)	0.013 (3)	0.010 (4)
C65	0.098 (6)	0.097 (7)	0.044 (4)	−0.001 (5)	0.034 (4)	−0.001 (5)
C66	0.112 (6)	0.082 (6)	0.056 (5)	0.004 (5)	0.039 (4)	−0.015 (4)
C67	0.070 (5)	0.058 (5)	0.060 (4)	0.003 (4)	0.017 (4)	−0.003 (4)
C68	0.051 (4)	0.049 (4)	0.038 (3)	−0.009 (3)	0.013 (3)	0.003 (3)
C69	0.069 (5)	0.033 (4)	0.067 (4)	−0.007 (3)	0.012 (4)	0.006 (3)
C70	0.063 (5)	0.067 (6)	0.075 (5)	−0.008 (4)	0.002 (4)	0.020 (4)
C71	0.056 (4)	0.071 (6)	0.070 (5)	−0.020 (4)	0.004 (4)	−0.006 (4)
C72	0.069 (5)	0.057 (5)	0.105 (6)	−0.017 (4)	−0.018 (5)	0.015 (5)
C73	0.077 (5)	0.042 (5)	0.082 (5)	−0.014 (4)	−0.009 (4)	−0.001 (4)
C74	0.048 (4)	0.045 (4)	0.056 (4)	−0.010 (4)	0.021 (3)	0.005 (3)
C75	0.044 (4)	0.077 (6)	0.062 (4)	−0.003 (4)	0.029 (3)	0.008 (4)
C76	0.076 (5)	0.065 (5)	0.119 (6)	−0.019 (4)	0.066 (5)	−0.003 (5)
C77	0.157 (10)	0.092 (8)	0.182 (10)	−0.025 (7)	0.111 (8)	0.004 (7)
C78	0.117 (8)	0.156 (12)	0.150 (10)	−0.020 (8)	0.093 (7)	0.022 (9)
C79	0.086 (6)	0.133 (8)	0.103 (6)	0.019 (6)	0.066 (5)	0.007 (6)
C80	0.067 (4)	0.076 (5)	0.069 (4)	0.011 (4)	0.038 (4)	0.016 (4)

### Geometric parameters (Å, º)

**Table:**

O1—C5	1.323 (8)	F2—C65	1.359 (7)
O1—C4	1.496 (7)	O6—C45	1.339 (7)
O2—C5	1.205 (7)	O6—C44	1.472 (8)
O3—C10	1.414 (7)	O7—C45	1.204 (7)
O3—C7	1.448 (6)	O8—C47	1.425 (6)
O4—C10	1.414 (7)	O8—C50	1.438 (7)
O4—C9	1.443 (6)	O9—C50	1.436 (7)
O5—C34	1.241 (7)	O9—C49	1.439 (6)
F1—C25	1.373 (7)	O10—C74	1.205 (7)
N1—C18	1.383 (7)	N3—C58	1.391 (7)
N1—C15	1.385 (6)	N3—C55	1.406 (6)
N1—C14	1.486 (7)	N3—C54	1.468 (6)
N2—C34	1.367 (7)	N4—C74	1.330 (7)
N2—C35	1.420 (7)	N4—C75	1.408 (7)
N2—H2A	0.8600	N4—H4A	0.8600
C1—C4	1.477 (9)	C41—C44	1.521 (9)
C1—H1A	0.9600	C41—H41A	0.9600
C1—H1B	0.9600	C41—H41B	0.9600
C1—H1C	0.9600	C41—H41C	0.9600
C2—C4	1.518 (9)	C42—C44	1.500 (9)
C2—H2B	0.9600	C42—H42A	0.9600
C2—H2C	0.9600	C42—H42B	0.9600
C2—H2D	0.9600	C42—H42C	0.9600
C3—C4	1.496 (9)	C43—C44	1.535 (9)
C3—H3A	0.9600	C43—H43A	0.9600
C3—H3B	0.9600	C43—H43B	0.9600
C3—H3C	0.9600	C43—H43C	0.9600
C5—C6	1.497 (8)	C45—C46	1.498 (8)
C6—C7	1.538 (7)	C46—C47	1.504 (7)
C6—H6A	0.9700	C46—H46A	0.9700
C6—H6B	0.9700	C46—H46B	0.9700
C7—C8	1.518 (7)	C47—C48	1.501 (6)
C7—H7A	0.9800	C47—H47A	0.9800
C8—C9	1.522 (7)	C48—C49	1.521 (7)
C8—H8A	0.9700	C48—H48A	0.9700
C8—H8B	0.9700	C48—H48B	0.9700
C9—C13	1.511 (7)	C49—C53	1.512 (7)
C9—H9A	0.9800	C49—H49A	0.9800
C10—C12	1.518 (8)	C50—C52	1.501 (8)
C10—C11	1.531 (7)	C50—C51	1.510 (9)
C11—H11A	0.9600	C51—H51A	0.9600
C11—H11B	0.9600	C51—H51B	0.9600
C11—H11C	0.9600	C51—H51C	0.9600
C12—H12A	0.9600	C52—H52A	0.9600
C12—H12B	0.9600	C52—H52B	0.9600
C12—H12C	0.9600	C52—H52C	0.9600
C13—C14	1.507 (8)	C53—C54	1.498 (8)
C13—H13A	0.9700	C53—H53A	0.9700
C13—H13B	0.9700	C53—H53B	0.9700
C14—H14A	0.9700	C54—H54A	0.9700
C14—H14B	0.9700	C54—H54B	0.9700
C15—C16	1.375 (7)	C55—C56	1.386 (7)
C15—C22	1.477 (7)	C55—C62	1.456 (7)
C16—C17	1.410 (7)	C56—C57	1.433 (7)
C16—C28	1.473 (7)	C56—C68	1.481 (7)
C17—C18	1.370 (7)	C57—C58	1.360 (7)
C17—C34	1.487 (8)	C57—C74	1.525 (8)
C18—C19	1.500 (7)	C58—C59	1.491 (7)
C19—C20	1.514 (9)	C59—C61	1.510 (8)
C19—C21	1.544 (9)	C59—C60	1.552 (9)
C19—H19A	0.9800	C59—H59A	0.9800
C20—H20A	0.9600	C60—H60A	0.9600
C20—H20B	0.9600	C60—H60B	0.9600
C20—H20C	0.9600	C60—H60C	0.9600
C21—H21A	0.9600	C61—H61A	0.9600
C21—H21B	0.9600	C61—H61B	0.9600
C21—H21C	0.9600	C61—H61C	0.9600
C22—C27	1.392 (8)	C62—C67	1.393 (8)
C22—C23	1.393 (8)	C62—C63	1.404 (8)
C23—C24	1.367 (7)	C63—C64	1.361 (7)
C23—H23A	0.9300	C63—H63A	0.9300
C24—C25	1.350 (9)	C64—C65	1.363 (9)
C24—H24A	0.9300	C64—H64A	0.9300
C25—C26	1.350 (10)	C65—C66	1.396 (10)
C26—C27	1.382 (8)	C66—C67	1.377 (9)
C26—H26A	0.9300	C66—H66A	0.9300
C27—H27A	0.9300	C67—H67A	0.9300
C28—C33	1.367 (8)	C68—C69	1.378 (8)
C28—C29	1.379 (8)	C68—C73	1.401 (8)
C29—C30	1.384 (7)	C69—C70	1.375 (8)
C29—H29A	0.9300	C69—H69A	0.9300
C30—C31	1.369 (9)	C70—C71	1.384 (9)
C30—H30A	0.9300	C70—H70A	0.9300
C31—C32	1.369 (8)	C71—C72	1.386 (8)
C31—H31A	0.9300	C71—H71A	0.9300
C32—C33	1.379 (8)	C72—C73	1.378 (8)
C32—H32A	0.9300	C72—H72A	0.9300
C33—H33A	0.9300	C73—H73A	0.9300
C35—C40	1.371 (8)	C75—C76	1.363 (9)
C35—C36	1.372 (8)	C75—C80	1.411 (8)
C36—C37	1.383 (8)	C76—C77	1.407 (10)
C36—H36A	0.9300	C76—H76A	0.9300
C37—C38	1.371 (10)	C77—C78	1.368 (13)
C37—H37A	0.9300	C77—H77A	0.9300
C38—C39	1.378 (10)	C78—C79	1.354 (13)
C38—H38A	0.9300	C78—H78A	0.9300
C39—C40	1.405 (8)	C79—C80	1.356 (9)
C39—H39A	0.9300	C79—H79A	0.9300
C40—H40A	0.9300	C80—H80A	0.9300
			
C5—O1—C4	121.7 (5)	C45—O6—C44	121.6 (5)
C10—O3—C7	114.7 (4)	C47—O8—C50	114.8 (5)
C10—O4—C9	114.9 (4)	C50—O9—C49	114.6 (5)
C18—N1—C15	109.4 (5)	C58—N3—C55	109.4 (4)
C18—N1—C14	125.8 (4)	C58—N3—C54	125.2 (4)
C15—N1—C14	124.3 (4)	C55—N3—C54	124.9 (4)
C34—N2—C35	129.1 (5)	C74—N4—C75	125.8 (6)
C34—N2—H2A	115.5	C74—N4—H4A	117.1
C35—N2—H2A	115.5	C75—N4—H4A	117.1
C4—C1—H1A	109.5	C44—C41—H41A	109.5
C4—C1—H1B	109.5	C44—C41—H41B	109.5
H1A—C1—H1B	109.5	H41A—C41—H41B	109.5
C4—C1—H1C	109.5	C44—C41—H41C	109.5
H1A—C1—H1C	109.5	H41A—C41—H41C	109.5
H1B—C1—H1C	109.5	H41B—C41—H41C	109.5
C4—C2—H2B	109.5	C44—C42—H42A	109.5
C4—C2—H2C	109.5	C44—C42—H42B	109.5
H2B—C2—H2C	109.5	H42A—C42—H42B	109.5
C4—C2—H2D	109.5	C44—C42—H42C	109.5
H2B—C2—H2D	109.5	H42A—C42—H42C	109.5
H2C—C2—H2D	109.5	H42B—C42—H42C	109.5
C4—C3—H3A	109.5	C44—C43—H43A	109.5
C4—C3—H3B	109.5	C44—C43—H43B	109.5
H3A—C3—H3B	109.5	H43A—C43—H43B	109.5
C4—C3—H3C	109.5	C44—C43—H43C	109.5
H3A—C3—H3C	109.5	H43A—C43—H43C	109.5
H3B—C3—H3C	109.5	H43B—C43—H43C	109.5
C1—C4—O1	109.8 (6)	O6—C44—C42	110.4 (6)
C1—C4—C3	114.4 (7)	O6—C44—C41	109.1 (6)
O1—C4—C3	109.2 (6)	C42—C44—C41	114.5 (7)
C1—C4—C2	109.8 (6)	O6—C44—C43	101.5 (6)
O1—C4—C2	101.7 (5)	C42—C44—C43	110.0 (7)
C3—C4—C2	111.0 (6)	C41—C44—C43	110.7 (7)
O2—C5—O1	124.5 (6)	O7—C45—O6	124.5 (6)
O2—C5—C6	124.2 (7)	O7—C45—C46	123.7 (7)
O1—C5—C6	111.3 (6)	O6—C45—C46	111.7 (6)
C5—C6—C7	109.3 (5)	C45—C46—C47	110.4 (5)
C5—C6—H6A	109.8	C45—C46—H46A	109.6
C7—C6—H6A	109.8	C47—C46—H46A	109.6
C5—C6—H6B	109.8	C45—C46—H46B	109.6
C7—C6—H6B	109.8	C47—C46—H46B	109.6
H6A—C6—H6B	108.3	H46A—C46—H46B	108.1
O3—C7—C8	109.1 (4)	O8—C47—C48	109.4 (4)
O3—C7—C6	106.1 (4)	O8—C47—C46	105.7 (5)
C8—C7—C6	112.8 (5)	C48—C47—C46	113.1 (5)
O3—C7—H7A	109.6	O8—C47—H47A	109.5
C8—C7—H7A	109.6	C48—C47—H47A	109.5
C6—C7—H7A	109.6	C46—C47—H47A	109.5
C7—C8—C9	109.3 (5)	C47—C48—C49	111.6 (5)
C7—C8—H8A	109.8	C47—C48—H48A	109.3
C9—C8—H8A	109.8	C49—C48—H48A	109.3
C7—C8—H8B	109.8	C47—C48—H48B	109.3
C9—C8—H8B	109.8	C49—C48—H48B	109.3
H8A—C8—H8B	108.3	H48A—C48—H48B	108.0
O4—C9—C13	107.2 (4)	O9—C49—C53	106.4 (5)
O4—C9—C8	109.1 (4)	O9—C49—C48	109.4 (5)
C13—C9—C8	111.4 (5)	C53—C49—C48	110.5 (5)
O4—C9—H9A	109.7	O9—C49—H49A	110.2
C13—C9—H9A	109.7	C53—C49—H49A	110.2
C8—C9—H9A	109.7	C48—C49—H49A	110.2
O3—C10—O4	110.9 (5)	O9—C50—O8	108.4 (5)
O3—C10—C12	110.7 (5)	O9—C50—C52	111.7 (5)
O4—C10—C12	113.4 (5)	O8—C50—C52	112.3 (5)
O3—C10—C11	104.3 (5)	O9—C50—C51	106.7 (6)
O4—C10—C11	106.7 (5)	O8—C50—C51	107.1 (6)
C12—C10—C11	110.5 (6)	C52—C50—C51	110.5 (6)
C10—C11—H11A	109.5	C50—C51—H51A	109.5
C10—C11—H11B	109.5	C50—C51—H51B	109.5
H11A—C11—H11B	109.5	H51A—C51—H51B	109.5
C10—C11—H11C	109.5	C50—C51—H51C	109.5
H11A—C11—H11C	109.5	H51A—C51—H51C	109.5
H11B—C11—H11C	109.5	H51B—C51—H51C	109.5
C10—C12—H12A	109.5	C50—C52—H52A	109.5
C10—C12—H12B	109.5	C50—C52—H52B	109.5
H12A—C12—H12B	109.5	H52A—C52—H52B	109.5
C10—C12—H12C	109.5	C50—C52—H52C	109.5
H12A—C12—H12C	109.5	H52A—C52—H52C	109.5
H12B—C12—H12C	109.5	H52B—C52—H52C	109.5
C14—C13—C9	113.3 (6)	C54—C53—C49	115.6 (5)
C14—C13—H13A	108.9	C54—C53—H53A	108.4
C9—C13—H13A	108.9	C49—C53—H53A	108.4
C14—C13—H13B	108.9	C54—C53—H53B	108.4
C9—C13—H13B	108.9	C49—C53—H53B	108.4
H13A—C13—H13B	107.7	H53A—C53—H53B	107.4
N1—C14—C13	113.2 (5)	N3—C54—C53	113.5 (5)
N1—C14—H14A	108.9	N3—C54—H54A	108.9
C13—C14—H14A	108.9	C53—C54—H54A	108.9
N1—C14—H14B	108.9	N3—C54—H54B	108.9
C13—C14—H14B	108.9	C53—C54—H54B	108.9
H14A—C14—H14B	107.8	H54A—C54—H54B	107.7
C16—C15—N1	107.6 (5)	C56—C55—N3	106.7 (5)
C16—C15—C22	129.4 (5)	C56—C55—C62	129.3 (5)
N1—C15—C22	122.5 (5)	N3—C55—C62	123.9 (5)
C15—C16—C17	107.3 (5)	C55—C56—C57	107.6 (5)
C15—C16—C28	125.2 (5)	C55—C56—C68	125.8 (5)
C17—C16—C28	127.3 (5)	C57—C56—C68	126.4 (5)
C18—C17—C16	108.8 (5)	C58—C57—C56	108.5 (5)
C18—C17—C34	127.8 (5)	C58—C57—C74	127.6 (5)
C16—C17—C34	123.4 (5)	C56—C57—C74	123.9 (5)
C17—C18—N1	107.0 (5)	C57—C58—N3	107.9 (5)
C17—C18—C19	132.0 (5)	C57—C58—C59	130.5 (5)
N1—C18—C19	120.6 (5)	N3—C58—C59	121.5 (5)
C18—C19—C20	111.7 (5)	C58—C59—C61	114.4 (6)
C18—C19—C21	111.8 (5)	C58—C59—C60	110.3 (5)
C20—C19—C21	109.9 (5)	C61—C59—C60	110.7 (5)
C18—C19—H19A	107.8	C58—C59—H59A	107.0
C20—C19—H19A	107.8	C61—C59—H59A	107.0
C21—C19—H19A	107.8	C60—C59—H59A	107.0
C19—C20—H20A	109.5	C59—C60—H60A	109.5
C19—C20—H20B	109.5	C59—C60—H60B	109.5
H20A—C20—H20B	109.5	H60A—C60—H60B	109.5
C19—C20—H20C	109.5	C59—C60—H60C	109.5
H20A—C20—H20C	109.5	H60A—C60—H60C	109.5
H20B—C20—H20C	109.5	H60B—C60—H60C	109.5
C19—C21—H21A	109.5	C59—C61—H61A	109.5
C19—C21—H21B	109.5	C59—C61—H61B	109.5
H21A—C21—H21B	109.5	H61A—C61—H61B	109.5
C19—C21—H21C	109.5	C59—C61—H61C	109.5
H21A—C21—H21C	109.5	H61A—C61—H61C	109.5
H21B—C21—H21C	109.5	H61B—C61—H61C	109.5
C27—C22—C23	117.5 (5)	C67—C62—C63	117.5 (5)
C27—C22—C15	119.0 (6)	C67—C62—C55	119.1 (6)
C23—C22—C15	123.4 (6)	C63—C62—C55	123.4 (6)
C24—C23—C22	121.9 (6)	C64—C63—C62	122.5 (6)
C24—C23—H23A	119.1	C64—C63—H63A	118.7
C22—C23—H23A	119.1	C62—C63—H63A	118.7
C25—C24—C23	118.4 (7)	C63—C64—C65	117.9 (7)
C25—C24—H24A	120.8	C63—C64—H64A	121.1
C23—C24—H24A	120.8	C65—C64—H64A	121.1
C26—C25—C24	122.3 (6)	F2—C65—C64	119.6 (8)
C26—C25—F1	118.9 (8)	F2—C65—C66	117.7 (7)
C24—C25—F1	118.7 (8)	C64—C65—C66	122.7 (7)
C25—C26—C27	119.8 (7)	C67—C66—C65	118.1 (7)
C25—C26—H26A	120.1	C67—C66—H66A	120.9
C27—C26—H26A	120.1	C65—C66—H66A	120.9
C26—C27—C22	119.8 (7)	C66—C67—C62	121.1 (7)
C26—C27—H27A	120.1	C66—C67—H67A	119.4
C22—C27—H27A	120.1	C62—C67—H67A	119.4
C33—C28—C29	116.4 (6)	C69—C68—C73	118.5 (6)
C33—C28—C16	122.9 (6)	C69—C68—C56	119.8 (6)
C29—C28—C16	120.6 (6)	C73—C68—C56	121.6 (6)
C28—C29—C30	122.3 (7)	C70—C69—C68	121.1 (6)
C28—C29—H29A	118.8	C70—C69—H69A	119.5
C30—C29—H29A	118.8	C68—C69—H69A	119.5
C31—C30—C29	119.9 (7)	C69—C70—C71	120.7 (7)
C31—C30—H30A	120.1	C69—C70—H70A	119.6
C29—C30—H30A	120.1	C71—C70—H70A	119.6
C30—C31—C32	118.6 (7)	C70—C71—C72	118.5 (7)
C30—C31—H31A	120.7	C70—C71—H71A	120.8
C32—C31—H31A	120.7	C72—C71—H71A	120.8
C31—C32—C33	120.6 (7)	C73—C72—C71	121.2 (7)
C31—C32—H32A	119.7	C73—C72—H72A	119.4
C33—C32—H32A	119.7	C71—C72—H72A	119.4
C28—C33—C32	122.1 (6)	C72—C73—C68	119.9 (7)
C28—C33—H33A	119.0	C72—C73—H73A	120.0
C32—C33—H33A	119.0	C68—C73—H73A	120.0
O5—C34—N2	121.7 (6)	O10—C74—N4	124.5 (6)
O5—C34—C17	122.9 (6)	O10—C74—C57	121.4 (6)
N2—C34—C17	115.4 (5)	N4—C74—C57	114.1 (6)
C40—C35—C36	120.0 (6)	C76—C75—N4	123.2 (7)
C40—C35—N2	116.4 (6)	C76—C75—C80	119.7 (6)
C36—C35—N2	123.5 (6)	N4—C75—C80	117.1 (6)
C35—C36—C37	120.3 (7)	C75—C76—C77	119.4 (8)
C35—C36—H36A	119.8	C75—C76—H76A	120.3
C37—C36—H36A	119.8	C77—C76—H76A	120.3
C38—C37—C36	121.2 (8)	C78—C77—C76	119.1 (9)
C38—C37—H37A	119.4	C78—C77—H77A	120.5
C36—C37—H37A	119.4	C76—C77—H77A	120.5
C37—C38—C39	118.0 (7)	C79—C78—C77	121.7 (10)
C37—C38—H38A	121.0	C79—C78—H78A	119.2
C39—C38—H38A	121.0	C77—C78—H78A	119.2
C38—C39—C40	121.6 (7)	C78—C79—C80	120.1 (9)
C38—C39—H39A	119.2	C78—C79—H79A	119.9
C40—C39—H39A	119.2	C80—C79—H79A	119.9
C35—C40—C39	118.8 (7)	C79—C80—C75	120.0 (8)
C35—C40—H40A	120.6	C79—C80—H80A	120.0
C39—C40—H40A	120.6	C75—C80—H80A	120.0
			
C5—O1—C4—C1	−62.9 (8)	C45—O6—C44—C42	−59.2 (8)
C5—O1—C4—C3	63.3 (8)	C45—O6—C44—C41	67.4 (8)
C5—O1—C4—C2	−179.3 (5)	C45—O6—C44—C43	−175.7 (6)
C4—O1—C5—O2	3.0 (10)	C44—O6—C45—O7	−3.1 (10)
C4—O1—C5—C6	−177.5 (5)	C44—O6—C45—C46	172.4 (6)
O2—C5—C6—C7	−114.3 (7)	O7—C45—C46—C47	69.0 (8)
O1—C5—C6—C7	66.2 (6)	O6—C45—C46—C47	−106.5 (6)
C10—O3—C7—C8	56.0 (6)	C50—O8—C47—C48	56.8 (6)
C10—O3—C7—C6	177.8 (5)	C50—O8—C47—C46	178.8 (4)
C5—C6—C7—O3	46.2 (6)	C45—C46—C47—O8	52.7 (7)
C5—C6—C7—C8	165.5 (5)	C45—C46—C47—C48	172.4 (5)
O3—C7—C8—C9	−54.3 (6)	O8—C47—C48—C49	−51.4 (6)
C6—C7—C8—C9	−171.9 (5)	C46—C47—C48—C49	−168.9 (5)
C10—O4—C9—C13	−176.5 (5)	C50—O9—C49—C53	−174.3 (5)
C10—O4—C9—C8	−55.7 (6)	C50—O9—C49—C48	−54.9 (6)
C7—C8—C9—O4	54.2 (6)	C47—C48—C49—O9	50.6 (6)
C7—C8—C9—C13	172.4 (5)	C47—C48—C49—C53	167.4 (5)
C7—O3—C10—O4	−55.2 (6)	C49—O9—C50—O8	57.7 (6)
C7—O3—C10—C12	71.5 (6)	C49—O9—C50—C52	−66.6 (7)
C7—O3—C10—C11	−169.7 (5)	C49—O9—C50—C51	172.7 (5)
C9—O4—C10—O3	55.2 (6)	C47—O8—C50—O9	−58.7 (6)
C9—O4—C10—C12	−70.0 (6)	C47—O8—C50—C52	65.1 (7)
C9—O4—C10—C11	168.2 (5)	C47—O8—C50—C51	−173.5 (5)
O4—C9—C13—C14	−69.7 (6)	O9—C49—C53—C54	−47.4 (7)
C8—C9—C13—C14	171.0 (5)	C48—C49—C53—C54	−166.1 (6)
C18—N1—C14—C13	−101.0 (6)	C58—N3—C54—C53	112.1 (6)
C15—N1—C14—C13	70.0 (7)	C55—N3—C54—C53	−58.3 (8)
C9—C13—C14—N1	−165.0 (4)	C49—C53—C54—N3	−161.4 (5)
C18—N1—C15—C16	−1.9 (7)	C58—N3—C55—C56	0.1 (6)
C14—N1—C15—C16	−174.1 (5)	C54—N3—C55—C56	171.8 (5)
C18—N1—C15—C22	170.2 (6)	C58—N3—C55—C62	−177.3 (6)
C14—N1—C15—C22	−2.0 (9)	C54—N3—C55—C62	−5.6 (9)
N1—C15—C16—C17	1.3 (7)	N3—C55—C56—C57	−0.6 (6)
C22—C15—C16—C17	−170.1 (6)	C62—C55—C56—C57	176.6 (6)
N1—C15—C16—C28	−173.4 (5)	N3—C55—C56—C68	173.8 (6)
C22—C15—C16—C28	15.2 (10)	C62—C55—C56—C68	−9.0 (10)
C15—C16—C17—C18	−0.3 (7)	C55—C56—C57—C58	0.9 (7)
C28—C16—C17—C18	174.3 (6)	C68—C56—C57—C58	−173.5 (6)
C15—C16—C17—C34	178.1 (5)	C55—C56—C57—C74	−179.0 (6)
C28—C16—C17—C34	−7.3 (10)	C68—C56—C57—C74	6.6 (10)
C16—C17—C18—N1	−0.9 (7)	C56—C57—C58—N3	−0.8 (7)
C34—C17—C18—N1	−179.2 (6)	C74—C57—C58—N3	179.1 (6)
C16—C17—C18—C19	−173.1 (6)	C56—C57—C58—C59	175.1 (6)
C34—C17—C18—C19	8.7 (11)	C74—C57—C58—C59	−5.0 (11)
C15—N1—C18—C17	1.7 (7)	C55—N3—C58—C57	0.4 (7)
C14—N1—C18—C17	173.8 (5)	C54—N3—C58—C57	−171.3 (5)
C15—N1—C18—C19	175.0 (5)	C55—N3—C58—C59	−175.9 (5)
C14—N1—C18—C19	−12.9 (9)	C54—N3—C58—C59	12.4 (9)
C17—C18—C19—C20	94.3 (8)	C57—C58—C59—C61	27.6 (10)
N1—C18—C19—C20	−77.0 (7)	N3—C58—C59—C61	−157.0 (6)
C17—C18—C19—C21	−29.3 (10)	C57—C58—C59—C60	−98.0 (8)
N1—C18—C19—C21	159.3 (6)	N3—C58—C59—C60	77.5 (7)
C16—C15—C22—C27	45.3 (9)	C56—C55—C62—C67	−46.7 (9)
N1—C15—C22—C27	−124.9 (6)	N3—C55—C62—C67	130.1 (6)
C16—C15—C22—C23	−133.7 (7)	C56—C55—C62—C63	133.8 (7)
N1—C15—C22—C23	56.0 (8)	N3—C55—C62—C63	−49.4 (8)
C27—C22—C23—C24	5.0 (9)	C67—C62—C63—C64	−4.0 (9)
C15—C22—C23—C24	−175.9 (5)	C55—C62—C63—C64	175.6 (5)
C22—C23—C24—C25	−3.2 (9)	C62—C63—C64—C65	2.0 (9)
C23—C24—C25—C26	−0.3 (11)	C63—C64—C65—F2	180.0 (6)
C23—C24—C25—F1	180.0 (6)	C63—C64—C65—C66	1.0 (11)
C24—C25—C26—C27	1.7 (13)	F2—C65—C66—C67	179.1 (6)
F1—C25—C26—C27	−178.5 (6)	C64—C65—C66—C67	−1.9 (13)
C25—C26—C27—C22	0.3 (12)	C65—C66—C67—C62	−0.2 (11)
C23—C22—C27—C26	−3.5 (9)	C63—C62—C67—C66	3.0 (9)
C15—C22—C27—C26	177.4 (6)	C55—C62—C67—C66	−176.6 (6)
C15—C16—C28—C33	−132.7 (7)	C55—C56—C68—C69	−55.3 (9)
C17—C16—C28—C33	53.7 (9)	C57—C56—C68—C69	118.1 (7)
C15—C16—C28—C29	49.7 (9)	C55—C56—C68—C73	127.7 (7)
C17—C16—C28—C29	−123.9 (7)	C57—C56—C68—C73	−58.9 (10)
C33—C28—C29—C30	2.3 (10)	C73—C68—C69—C70	−4.2 (10)
C16—C28—C29—C30	−180.0 (6)	C56—C68—C69—C70	178.7 (6)
C28—C29—C30—C31	−1.2 (10)	C68—C69—C70—C71	2.7 (11)
C29—C30—C31—C32	−1.3 (11)	C69—C70—C71—C72	−0.4 (12)
C30—C31—C32—C33	2.5 (11)	C70—C71—C72—C73	−0.3 (12)
C29—C28—C33—C32	−1.1 (11)	C71—C72—C73—C68	−1.2 (13)
C16—C28—C33—C32	−178.7 (6)	C69—C68—C73—C72	3.4 (11)
C31—C32—C33—C28	−1.3 (12)	C56—C68—C73—C72	−179.5 (7)
C35—N2—C34—O5	0.4 (10)	C75—N4—C74—O10	2.6 (10)
C35—N2—C34—C17	−179.9 (5)	C75—N4—C74—C57	−179.2 (5)
C18—C17—C34—O5	89.5 (9)	C58—C57—C74—O10	−97.0 (9)
C16—C17—C34—O5	−88.5 (8)	C56—C57—C74—O10	82.9 (9)
C18—C17—C34—N2	−90.2 (7)	C58—C57—C74—N4	84.7 (8)
C16—C17—C34—N2	91.8 (7)	C56—C57—C74—N4	−95.4 (7)
C34—N2—C35—C40	−178.1 (5)	C74—N4—C75—C76	−29.9 (10)
C34—N2—C35—C36	2.1 (9)	C74—N4—C75—C80	147.5 (6)
C40—C35—C36—C37	1.0 (10)	N4—C75—C76—C77	−179.5 (7)
N2—C35—C36—C37	−179.3 (5)	C80—C75—C76—C77	3.2 (11)
C35—C36—C37—C38	0.5 (11)	C75—C76—C77—C78	−2.6 (14)
C36—C37—C38—C39	−1.3 (13)	C76—C77—C78—C79	2.0 (17)
C37—C38—C39—C40	0.8 (13)	C77—C78—C79—C80	−2.1 (17)
C36—C35—C40—C39	−1.5 (9)	C78—C79—C80—C75	2.7 (12)
N2—C35—C40—C39	178.8 (5)	C76—C75—C80—C79	−3.3 (10)
C38—C39—C40—C35	0.6 (12)	N4—C75—C80—C79	179.2 (6)

### Hydrogen-bond geometry (Å, º)

**Table:**

*D*—H···*A*	*D*—H	H···*A*	*D*···*A*	*D*—H···*A*
N2—H2*A*···O2^i^	0.86	2.05	2.899 (7)	168
N4—H4*A*···O7^ii^	0.86	2.07	2.914 (7)	168

Symmetry codes: (i) *x*+1, *y*, *z*; (ii) *x*−1, *y*, *z*.
